# Systemic lupus Erythematosus activity and Hydroxychloroquine use before and after end-stage renal disease

**DOI:** 10.1186/s12882-020-02083-2

**Published:** 2020-10-28

**Authors:** Maria Salgado Guerrero, Alejandra Londono Jimenez, Chrisanna Dobrowolski, Wenzhu B. Mowrey, Beatrice Goilav, Shudan Wang, Anna Broder

**Affiliations:** 1 Department of Medicine, Jacobi Medical Center/Albert Einstein College of Medicine, Bronx, New York USA; 2 Division of Rheumatology, Department of Medicine, Montefiore Medical Center/Albert Einstein College of Medicine, Bronx, New York USA; 3 Division of Biostatistics, Department of Epidemiology and Population Health, Albert Einstein College of Medicine, Bronx, New York USA; 4 Division of Pediatric Nephrology, Department of Pediatrics, Montefiore Medical Center/Albert Einstein College of Medicine, Bronx, New York USA

**Keywords:** Disease activity, Systemic lupus Erythematosus, End-stage renal disease, Hydroxychloroquine

## Abstract

**Background:**

SLE manifestations after ESRD may be underdiagnosed and undertreated, contributing to increased morbidity and mortality. Whether specific symptoms persist after ESRD or a shift towards new manifestations occurs has not been extensively studied, especially in the non-Caucasian patients in the United States. In addition, hydroxychloroquine (HCQ) prescribing patterns post-ESRD have not been described. The objective of this study was to assess lupus activity and HCQ prescribing before and after ESRD development. Knowledge gained from this study may aid in the identification of SLE manifestations and improve medication management post-ESRD.

**Methods:**

We performed a retrospective cohort study of SLE patients with incident ESRD between 2010 and 2017. SLE-related symptoms, serologic markers of disease activity, and medication use were collected from medical records before and after ESRD development.

**Results:**

Fifty-nine patients were included in the study. Twenty-five (43%) patients had at least one clinical (non-renal) SLE manifestation documented within 12 months before ESRD. Of them, 11/25 (44%) continued to experience lupus symptoms post-ESRD; 9 patients without clinical or serological activity pre-ESRD developed new symptoms of active SLE. At the last documented visit post-ESRD, 42/59 (71%) patients had one or more clinical or serological markers of lupus activity; only 17/59 (29%) patients achieved clinical and serological remission.

Thirty-three of 59 (56%) patients had an active HCQ prescription at the time of ESRD. Twenty-six of the 42 (62%) patients with active SLE manifestations post-ESRD were on HCQ. Patients who continued HCQ post-ESRD were more likely to be followed by a rheumatologist (26 [87%] vs 17 [61%], *p* = 0.024), had a higher frequency of documented arthritis (10 [32%] vs 1 [4%], *p* = 0.005), CNS manifestations (6 [20%] vs 1 [4%], *p* = 0.055), and concurrent immunosuppressive medication use (22 [71%] vs 12 [43%], *p* = 0.029).

**Conclusions:**

Lupus activity may persist after the development of ESRD. New onset arthritis, lupus-related rash, CNS manifestations, low complement and elevated anti-dsDNA may develop. HCQ may be underutilized in patients with evidence of active disease pre- and post ESRD. Careful clinical and serological monitoring for signs of active disease and frequent rheumatology follow up is advised in SLE patients both, pre and post-ESRD.

## Background

Lupus related end-stage renal disease (ESRD) is the most common complication of lupus nephritis (LN) [1]. The estimated mortality in patients with systemic lupus erythematosus (SLE) related ESRD is four-fold higher than in SLE patients with LN alone [2], and twice higher than non-SLE ESRD patients [3, 4].

SLE manifestations after ESRD may be underdiagnosed and undertreated, contributing to increased morbidity and mortality [5–7]. Diagnosing active SLE post-ESRD presents a major challenge. Patients who achieve clinical remission often stop following up with rheumatologists, making it difficult to monitor for subsequent flares. Some SLE manifestations, such as cytopenias, serositis, and seizures, are difficult to differentiate from complications of medication side effects or uremia [8–12]. Prior studies have shown that SLE activity can persist even after progression to ESRD [7, 13–17]; both clinical symptoms and signs of disease activity have been reported after the development of ESRD [5, 7, 11, 15]. However, most studies to date have focused on evaluating SLE disease activity after ESRD onset, rather than on assessing its evolution before and after ESRD [9, 11, 12, 18–25]. Understanding whether specific symptoms persist after ESRD or a shift towards new manifestations occurs may aid in the diagnosis of SLE manifestations post-ESRD and move towards individualized treatment decisions.

At present, there are no evidence-based guidelines on how to manage SLE related medications after ESRD [26–28]. Specifically, there is a great deal of uncertainty among rheumatologists and nephrologists on whether hydroxychloroquine (HCQ) should be continued once ESRD develops. HCQ is the cornerstone of treatment in SLE that has been associated with less SLE damage, fewer SLE flares and lower incidence of thrombosis [29–32]. However, these benefits are less well established among SLE patients with ESRD, especially because of the higher risk of HCQ toxicity in this patient population due to decreased renal clearance [33]. Understanding HCQ prescribing patterns and associated factors is a necessary first step towards the identification of patients who may benefit from continuing HCQ, with the ultimate goal of improving outcomes in some while decreasing unnecessary exposure and toxicity in others.

Therefore, the objective of this study was to 1) evaluate how both clinical and serological SLE activity evolves pre-and post-ESRD and 2) study the factors that may influence HCQ prescribing post-ESRD. Knowledge gained from this study may aid in the identification of SLE manifestations and HCQ usage post-ESRD.

## Methods

We conducted a retrospective chart review at Montefiore Medical Center (MMC), an academic community-based urban tertiary care center in The Bronx, New York [34]. Data was extracted from the electronic medical record using “Clinical Looking Glass” (CLG). CLG is a proprietary software application developed at Montefiore Medical Center that allows clinicians and researchers to identify populations of interest from the medical center database and to gather information such as laboratory data, medications, demographics, mortality, and other parameters [35]. This study was approved by the Institutional Review Board Committee (IRB# 2019–10,326) at Montefiore Medical Center, Albert Einstein College of Medicine.

All patients over the age of 18 years who had SLE and developed ESRD due to LN between 2010 and 2017 were identified. ESRD was defined as patients with glomerular filtration rate < 15 ml/min/1.73m^2^ who were on continuous dialysis for 6 months. Records of patients identified by this search were subsequently reviewed by two investigators (MS and AB) to identify patients who fulfilled either the American College of Rheumatology (ACR) criteria [36, 37] or the Systemic Lupus International Collaborating Clinics (SLICC) criteria for SLE [38]. Patients were excluded if they did not have evidence of LN on biopsy as the underlying disease leading to ESRD.

SLE manifestations, medication use, and laboratory values were collected before and after ESRD development. Pre-ESRD, data was collected from the visits documented within 12 months before ESRD. Post-ESRD, data was collected at any point after 1 month of ESRD because of the great variability with respect to visit frequency and duration of follow-up after initiation of dialysis. SLE manifestations and laboratory parameters were obtained from clinical documentation; arthritis (≥2 joints with pain and signs of inflammation), lupus-related rash, alopecia, oral ulcers, serositis (pericardial pain with ≥1 of the following: rub, effusion, or electrocardiogram/echocardiogram confirmation), central nervous system (CNS) manifestations reported as seizures (after careful chart review with neurology/nephrology assessment excluding uremia, metabolic and drug causes) and/or psychosis, complement levels (CH50, C3, or C4 decreased below lower limit of normal for lab), anti-dsDNA (Increased above normal range for lab), leukopenia (< 3000 white blood cell/mm3), and thrombocytopenia (< 100,000 platelets/mm3). Medications included: prednisone, azathioprine, cyclophosphamide, mycophenolate mofetil, tacrolimus, belimumab and rituximab. For patients who received a kidney transplant, information was collected only before transplantation.

Descriptive statistics were used to assess disease activity pre- and post-ESRD. Bivariate analyses were used to compare patients who were prescribed HCQ post-ESRD vs. patients who discontinued HCQ after ESRD developed. Wilcoxon-Mann-Whitney tests were used to compare non normally distributed continuous variables, while Pearson’s Chi-square tests (or Fisher’s exact test when appropriate) were used to compare categorical variables. *P* < 0.05 was considered statistically significant. Statistical analysis was performed using the STATA 12.0 (StataCorp LP, College Station, Texas).

## Results

Of the 59 patients included in the study, 48 (81%) were women, 25 (42%) were Hispanic or Latino, and 32 (54%) were Black or African American based on self-report. The median (IQR) age was 39 (26, 50) years old. The median time from SLE to ESRD diagnosis was 96 (36, 180) months. The median duration of follow-up from the first visit pre-ESRD to ESRD onset was 15 (7, 26) months, and the median duration follow-up from ESRD onset to the last visit post-ESRD was 32 (12, 62) months. The median number of rheumatology and nephrology visits pre-ESRD was 5 (2, 10), and 2 (1, 5) respectively, with a total number of pre-ESRD visits of 6 (4, 15). Post-ESRD, the median number of rheumatology and nephrology visits was 3 (1, 8) and 5 (2,10), with a total number of post-ESRD visits of 9 (5, 22).

### SLE disease activity

Twenty-five (43%) patients had at least one clinical (non-renal) criteria documented within 12 months before the development of ESRD (Table 1). Of them, 11/25 (44%) continued to experience persistent clinical symptoms post-ESRD: arthritis persisted in 4/13 (31%) of patients with pre-ESRD arthritis, rash in 3/7 (43%), CNS manifestations in 2/6 (33%), and recurrent alopecia in 2/8 (25%). Oral ulcers and serositis were reported in 3 and 5 patients pre-ESRD, respectively, but none had recurrence documented post-ESRD. Leukopenia and thrombocytopenia persisted in 12/18 (67%) patients and in 3/10 (30%) patients who had leukopenia and thrombocytopenia pre-ESRD. Anti-dsDNA levels remained elevated in 17 of 29 (59%) patients who had elevated anti-dsDNA pre-ESRD, and low complement levels persisted in 29 of 37 (78%) patients with low complement levels pre-ESRD.

**Table 1:** Clinical and Serological SLE-manifestations pre- and post-ESRD (*N* = 59)

	Present pre-ESRD	Persisted post-ESRD	New post-ESRD
Arthritis	13	4	7
Rash	7	3	3
Oral Ulcers	3	0	2
Alopecia	8	2	1
Central nervous system manifestations^a^	6	2	5
Serositis	5	0	3
Leukopenia	18	12	19
Thrombocytopenia	10	3	7
Low complement	37	29	3
Elevated anti-dsDNA	29	17	5

^a^Seizures and/or psychosis

Post-ESRD, a number of patients developed new SLE-related symptoms that were not reported pre-ESRD: 3 each developed new lupus-related rash and serositis; 7 developed arthritis; 2 developed oral ulcers; 1 developed alopecia; and 5 developed new CNS manifestations (seizures and/or psychosis). New onset leukopenia and thrombocytopenia post-ESRD were reported in 19 and 7 patients, respectively. Three patients developed low complement and 5 developed elevated anti-dsDNA post-ESRD. There was no association between clinical and serological activity post-ESRD. Serological activity was documented in 18 (46%) of patients without clinical symptoms and in 9 (47%) with clinical symptoms post-ESRD, *p*-value 0.93.

At the last documented post-ESRD visit, 42/59 (71%) patients had one or more clinical or serological markers of lupus activity; only 17/59 (29%) patients achieved clinical and serological remission. Patients with and without evidence of disease activity did not differ in their frequency of immunosuppressive medication use [22 (52%) vs 10 (59%), *p* = 0.65], nor in their rheumatology follow up after ESRD [32 (74%) vs 12 (71%), *p* = 0.80]. The median duration of follow up from ESRD onset to the last documented post-ESRD visit was similar for patients with and without symptoms or signs of active disease [32 (12, 62) months vs. 32 (11, 53) months, *p* = 0.96].

### Hydroxychloroquine prescribing patterns pre and post-ESRD

A total number of 24/41 (58%) patients with evidence of active disease were on HCQ pre-ESRD comparing with 26/42 (62%) patients with evidence of active disease post-ESRD.

Pre-ESRD, eighteen (44%) of the 41 patients with manifestations of active disease were taking HCQ and prednisone in combination with an immunosuppressive medication, 5 (12%) were on HCQ and prednisone only, 7 (17%) patients were taking prednisone in combination with an immunosuppressive medication. Five patients were on prednisone alone, 1 on HCQ only and 2 patients were only on immunosuppressive medication. Three patients with active disease were not taking any medication.

In contrast, post-ESRD, fourteen (33%) of the 42 patients with manifestations of active disease were taking HCQ and prednisone in combination with an immunosuppressive medication, 9 (21%) were taking HCQ and prednisone only, 6 (14%) were taking prednisone in combination with an immunosuppressive medication, 1 was taking HCQ in combination with an immunosuppressive medication. Four patients were on prednisone alone, 2 on HCQ only and 1 patient was only on immunosuppressive medication. Five patients were never prescribed SLE-related medications post-ESRD. Of the 17 patients with no manifestations of active disease post-ESRD, 7 (41%) were taking HCQ and prednisone in combination with an immunosuppressive medication, 2 (12%) were taking HCQ and prednisone only, 3 (18%) were taking prednisone in combination with an immunosuppressive medication. Two patients were on prednisone only, 1 patient was on HCQ only, and 2 were not taking any SLE related medications.

Hydroxychloroquine prescribing patterns post-ESRD onset are shown in Table 2. Of the 59 patients included in the study, 33 (56%) patients were taking HCQ at ESRD onset. Of them, 21 (64%) remained on HCQ at the last documented post-ESRD visit, and 12 (36%) discontinued HCQ. Eight patients initiated HCQ within 6 months post-ESRD. Therefore, 29/59 (49%) patients had an active HCQ prescription at the last post-ESRD visit. Patients taking HCQ were more likely to be followed by a rheumatologist (26 [87%] vs 17 [61%], *p* = 0.024), had a higher frequency of documented arthritis (10 [32%] vs 1 [4%], *p* = 0.005), CNS manifestations reported as seizures and/or psychosis (6 [20%] vs 1 [4%], *p* = 0.055), and a higher frequency of immunosuppressive medication use post ESRD (22 [71%] vs 12 [43%], *p* = 0.029) (Table 2). Patients receiving HCQ at the last post-ESRD visit were more likely to be Hispanic or Latino (16 [52%] vs 9 [32%], *p* = 0.315), and were younger compared with the patients not receiving HCQ (33 [26, 48] vs 47 [32, 54], *p* = 0.068). However, these associations were not statistically significant. There was no association between HCQ use and history of lupus-related rash, oral ulcers, alopecia, serositis, cytopenias, low complement or elevated dsDNA levels post-ESRD.

**Table 2:** Comparison of baseline demographics, and clinical/serological SLE-manifestations with HCQ use following ESRD diagnosis^a^

	HCQ use ***N*** = 31	No HCQ use ***N*** = 28	***p***-value
Age at ESRD onset, median (IQR), years	33 (26, 48)	47 (32, 54)	0.068
Race, n(%) Black or African-American Other	14 (45)	18 (64)	0.315
Hispanic or Latino, n(%)	16 (52)	9 (32)	0.315
Women, n(%)	25 (81)	23 (82)	0.883
Time from ESRD onset to last documented visit, median(IQR), months	33 (19, 60)	43 (9, 89)	0.564
Central nervous system manifestations, n(%)	6 (20)	1 (4)	0.055
Arthritis, n(%)	10 (32)	1 (4)	0.005
Rash, n(%)	4 (13)	2 (7)	0.439
Oral ulcers, n(%)	1 (4)	1 (4)	0.980
Alopecia, n(%)	1 (4)	2 (7)	0.532
Serositis, n(%)	1 (4)	2 (7)	0.532
Leukopenia, n(%)	17 (55)	17 (60)	0.648
Thrombocytopenia, n(%)	15 (48)	20 (71)	0.072
Low complement, n(%)	15 (54)	16 (57)	0.788
Elevated anti-dsDNA, n(%)	13 (46)	7 (25)	0.094
Corticosteroid use, n(%)	28 (90)	20 (74)	0.102
Immunosuppressive use, n(%)^b^	22 (71)	12 (43)	0.029
At least one rheumatology visit post-ESRD, n(%)	26 (87)	17 (61)	0.024
History of anti-phospholipid syndrome, n(%)	4 (13)	2 (7)	0.493
History of deep vein thrombosis, n(%)	4 (13)	3 (11)	0.834
Renal transplantation after ESRD, n(%)	10 (32)	7 (25)	0.539

^a^ SLE manifestations and medication use are included if they occurred at any time point after ESRD onset

^b^ Azathioprine, cyclophosphamide, mycophenolate mofetil, tacrolimus, belimumab and rituximab

At the last documented visit post-ESRD, 25/33 (76%) patients were taking HCQ 200 mg daily, and 8/33 (24%) were taking HCQ 400 mg daily. Of the 8 patients who were started on HCQ post-ESRD, 7 (88%) were started on HCQ 200 mg daily and only 1(12%) was started on HCQ 400 mg daily. At ESRD onset, 14/28 (50%) patients on HCQ were taking 200 mg daily and 14/28 (50%) were taking 400 mg daily. Of the 12 patients who discontinued HCQ post-ESRD, 1 (8%) had documented HCQ retinopathy on 200 mg HCQ daily, 1 (8%) reported dysphoria, 6 (50%) patients had “inactive SLE” and 4 (33%) had not documented reason for HCQ discontinuation.

Of the 42 patients with at least one SLE manifestation of active disease after ESRD, 26 (62%) were on HCQ. Of them, 20 (78%) had an active order of HCQ 200 mg daily and 6 (22%) had an active HCQ order of 400 mg daily. Of the 17 patients with no manifestations of active disease post-ESRD, 10 (59%) were on HCQ, 7(70%) of them were taking HCQ 200 mg daily and 3 (30%) were taking HCQ 400 mg daily.

## Discussion

In this study, we found that both clinical and serological activity was documented in most patients after the development of ESRD. At the last documented post-ESRD visit, 71% of patients had at least one or more clinical or serological marker of disease activity. This is consistent with prior studies that reported clinical and serological evidence of SLE-activity in 54–79% of patients after ESRD onset [5, 9, 13, 15]. The major novel finding of this study is that new SLE manifestations may develop after ESRD onset. New onset of arthritis, serositis, SLE-related rash, oral ulcers, CNS manifestations, leukopenia, thrombocytopenia, elevated anti-dsDNA and low complement levels were observed after ESRD development, suggesting that pre-ESRD signs and symptoms are not always predictive of post-ESRD disease course.

In our study, persistent serological activity was the most frequently reported finding after the ESRD development. Low complement levels and elevated anti-dsDNA levels remained abnormal in 78 and 59% of patients, respectively. This is comparable to the findings reported by Goo et al. [7] where 80% of patients remained serologically active during the first year of dialysis, and 52% remained serologically active 3 years later. Similarly, Kane at el [5] observed that lupus serological activity persisted in 57% of patients on hemodialysis within 3 years after ESRD onset. Arthritis, lupus-related rash, and CNS manifestations were the most common clinical manifestation observed after ESRD, consistent with previous reports [11, 20, 39].

Our results also demonstrate that “sicker” patients with arthritis and CNS symptoms who were on immunosuppression were more likely to be prescribed HCQ post-ESRD. However, 38% of patients with clinical symptoms of active SLE were not receiving HCQ post-ESRD, suggesting that HCQ may be underutilized in patients with evidence of active disease pre- and post-ESRD. Additionally, a significant number of patients (21%) with symptoms of active disease were treated with corticosteroids alone or were no taking any medication at all. Patients who continued to see a rheumatologist were also more likely to be prescribed HCQ.

It is recommended that HCQ dose should be reduced in ESRD due to decreased renal excretion in these patients and a concern for potential toxicity [40, 41]. In this relatively small sample of SLE ESRD patients, two patients had a documented HCQ related toxicity despite dose adjustment underscoring the need for further studies to examine the relationship of blood HCQ level, drug efficacy, and toxicity in SLE patients with and without ESRD.

This study has several limitations mainly related to its retrospective design and small sample size. A direct comparison of the medication utilization between patients with and without ESRD was not possible due to the biases associated with retrospective studies, including by-indication bias, selection bias, and due to significant differences with regards to disease activity and outcomes in ESRD and non-ESRD patients. Limited systemic evaluation and documentation by the different providers may have resulted in under-recognition of some SLE symptoms. Although systematic SLE assessments were not routinely performed for all patients, we were able to recreate the SLE disease activity index (SLEDAI) based on the available parameters of disease activity defined in the SLEDAI [42]. Our data were consistent with the previously reported studies [5, 7, 20] supporting the external validity of our findings.

The study may have been underpowered to detect some important differences between patients with and without HCQ prescriptions. The duration of follow-up, immunosuppressive medication use and the proportion of patients with rheumatologic follow-up were similar between patients with and without reported manifestations at the last post-ESRD visit, as well as the number of rheumatology and nephrology visits pre and post-ESRD, suggesting that lack of follow-up or monitoring was not the cause of the differences observed in this study. However, reporting bias cannot be completely accounted for in this retrospective study.

Despite these limitations, this study has some important strengths. SLE activity was analyzed before and after ESRD development providing a better comparison of the different SLE manifestations. Importantly, this study also reflects a “real-life” experience when ESRD patients receive fragmented care and their disease may be underdiagnosed and undertreated.

## Conclusion

In conclusion, lupus activity may persist after the development of ESRD. Additionally, new onset of arthritis, lupus-related rash, CNS manifestations, elevated anti-dsDNA, and low complement levels may develop. Therefore, careful clinical and serological monitoring for subtle signs of active disease and frequent rheumatology follow up is advised both, pre and post-ESRD. HCQ may be underutilized in patients with active disease. Further studies are needed to determine the risks and benefits of HCQ in SLE ESRD patients.

## Data Availability

The datasets analyzed in the current study is available from the corresponding author on reasonable request.

## Abbreviations

ESRD: End Stage Renal Disease
HCQ: Hydroxychloroquine
SLE: Systemic Lupus Erythematosus
CNS: Central Nervous System
LN: Lupus Nephritis
CLG: Clinical Looking Glass
MMC: Montefiore Medical Center
ACR: American College of Rheumatology
SLICC: Systemic Lupus International Collaborating Clinics
SLEDAI: Systemic Lupus Erythematosus Disease Activity Index
